# Virulence genes and subclone status as markers of experimental virulence in a murine sepsis model among *Escherichia coli* sequence type 131 clinical isolates from Spain

**DOI:** 10.1371/journal.pone.0188838

**Published:** 2017-11-30

**Authors:** Irene Merino, Stephen B. Porter, Brian D. Johnston, Connie Clabots, Evelyn Shaw, Juan Pablo Horcajada, Rafael Cantón, Patricia Ruiz-Garbajosa, James R. Johnson, for the ITUBRAS-GEIH group

**Affiliations:** 1 Servicio de Microbiología, Hospital Universitario Ramón y Cajal-Instituto Ramón y Cajal de Investigación Sanitaria (IRYCIS), Madrid, Spain; 2 Spanish Network for Research in Infectious Diseases (REIPI), Madrid, Spain; 3 Minneapolis Veterans Health Care System, Minneapolis, MN, United States of America; 4 University of Minnesota, Minneapolis, MN, United States of America; 5 Servicio de Enfermedades Infecciosas, Hospital Universitario de Bellvitge-IDIBELL, Barcelona, Spain; 6 Hospital del Mar-Medical Research Institute of Hospital del Mar (IMIM)-CEXS Universitat Pompeu Fabra, Barcelona, Spain; Universidad de Santiago de Compostela, SPAIN

## Abstract

**Objective:**

To assess experimental virulence among sequence type 131 (ST131) *Escherichia coli* bloodstream isolates in relation to virulence genotype and subclone.

**Methods:**

We analysed 48 Spanish ST131 bloodstream isolates (2010) by PCR for ST131 subclone status (*H*30Rx, *H*30 non-Rx, or non-*H*30), virulence genes (VGs), and O-type. Then we compared these traits with virulence in a murine sepsis model, as measured by illness severity score (ISS) and rapid lethality (mean ISS ≥ 4).

**Results:**

Of the 48 study isolates, 65% were *H*30Rx, 21% *H*30 non-Rx, and 15% non-*H*30; 44% produced ESBLs, 98% were O25b, and 83% qualified as extraintestinal pathogenic *E*. *coli* (ExPEC). Of 49 VGs, *ibeA* and *iss* were associated significantly with non-*H*30 isolates, and *sat*, *iha* and *malX* with *H*30 isolates. Median VG scores differed by subclone, i.e., 12 (*H*30Rx), 10 (*H*30 non-Rx), and 11 (non-*H*30) (p < 0.01). Nearly 80% of isolates represented a described virotype. In mice, *H*30Rx and non-*H*30 isolates were more virulent than *H*30 non-Rx isolates (according to ISS [p = 0.03] and rapid lethality [p = 0.03]), as were ExPEC isolates compared with non-ExPEC isolates (median ISS, 4.3 vs. 2.7: p = 0.03). In contrast, most individual VGs, VG scores, VG profiles, and virotypes were not associated with mouse virulence.

**Conclusions:**

ST131 subclone and ExPEC status, but not individual VGs, VG scores or profiles, or virotypes, predicted mouse virulence. Given the lower virulence of non-Rx *H*30 isolates, hypervirulence probably cannot explain the ST131-*H*30 clade's epidemic emergence.

## Introduction

The spread of a specific *Escherichia coli* clone called sequence type 131 (ST131) is one of the key drivers of the rising prevalence of resistance to quinolones and cephalosporins among *E*. *coli* that cause urinary tract infections, with cephalosporin resistance being mainly due to expression of extended-spectrum beta-lactamases (ESBLs), particularly CTX-M-15 [1–3]. The as-yet-unknown reasons for this globally distributed clone's epidemic success are under active investigation [4].

The ST131 pandemic is driven mainly by two nested subclones within ST131, i.e., *H*30 (or clade C), which contains allele 30 of *fimH* (type 1 fimbriae), and *H*30Rx (or clade C2), a sublineage within *H*30 that often expresses CTX-M-15 [4]. Strains that encode allele *H*30 but do not produce CTX-M-15 are usually grouped in the *H*30 non-Rx subclone (or Clade C1). Several studies have suggested that, apart from being extensively resistant, ST131 is also highly virulent, with a higher capability of causing infection than other strains [5,6]. ST131 virulence has been assessed in different animal models [7–9] with different conclusions. Moreover, these studies have not addressed ST131 subclone status.

ST131 usually contains a high number of putative virulence genes (VGs) which probably contributes to its virulence [7]. Indeed, in multiple studies, ST131 has been associated with specific VGs, including *usp* (uropathogenic specific protein), *iutA* (aerobactin receptor), *fyuA* (yersiniobactin receptor), *ompT* (outer membrane protein T), and *iroN* (salmochelin receptor) [10–14], which variously encode for toxins, adhesins, siderophores, or outer membrane proteins. Moreover, within ST131, based on specific combinations of VGs some authors have described virotypes A, B, C, D (with subsets 1–5), and E [10,15,16]. Of these virotypes, A, B and C have been associated with higher experimental virulence [15].

Hence, we sought to clarify these relationships among ST131 isolates from Spain by studying 48 ST131 isolates, including representatives of the different ST131 subclones. Specifically, we characterized our ST131 isolates extensively *in vitro*, assessed their virulence in a murine sepsis model, and sought correlations between experimental virulence and VG content, virotype, ExPEC status, and ST131 subclone (*H*30Rx, *H*30 non-Rx, and non-*H*30).

## Material and methods

### Isolates and detection of ST131 subclones and O types

The present ST131 study isolates represented a subset of the ITU-BRAS collection, the product of a Spanish multicentre study of bloodstream isolates from patients with bacteraemia of urinary origin who were admitted in 8 hospitals of different geographic areas from October 2010 to June 2011 [17]. This study was approved by the Ethics Commitee for Clinical Research of Hospital Ramón y Cajal. The collection's 425 *E*. *coli* isolates were characterized previously for ESBL production by PCR and sequencing [18]. Here, they were screened for ST131 status using PCR-based detection of ST131-specific single-nucleotide polymorphisms (SNPs) within *mdh* and *gyrB* [19]. ST131 isolates were further classified into the *H*30 and *H*30Rx subclones by PCR-based detection of subclone-specific SNPs [20] and were screened by PCR for the two most common O types found in ST131 strains, O25b and O16 [21]. For extended virulence genotyping and testing in the murine sepsis model we selected all ESBL-producing ST131 isolates (n = 21), plus 27 arbitrarily chosen non-ESBL-producing ST131 isolates, giving 48 isolates total. Moreover, to stablish the clonal relationship the 48 ST131 isolates were typed by PFGE-XbaI and a dendrogram was created using the PFGE-patterns (Bionumerics version 7.5, Applied Maths NV) according to the unweighted pair group mean arithmetic method based on pairwise Dice similarity coefficients.

### Virulence gene scores and virotypes

Forty-nine putative ExPEC-associated VGs and variants thereof were determined by an established multiplex PCR assay (Table 1) [10,22–24]. The VG score was calculated as the total number of VGs detected, adjusted for multiple detection of the *pap* (pilus associated with pyelonephritis, P fimbriae), *sfa/foc* (S and F1C fimbriae), and *kpsII* (capsular polysaccharide synthesis group 2) operons [25]. Isolates were classified as ExPEC if they carried ≥ 2 of *papAH* and/or *papC* (counted as one), *sfa/foc*, *afa/dra* (Dr antigen specific adhesin), *iutA*, and *kpsMII* [25].

**Table 1:** Distribution of virulence genes by ST131 subclones.

Virulence genes^a^^,^ ^b^		N° of isolates with gene^a^^,^^b^ (column %)				p value^c^	
Functional category	Specific gene	Total (n = 48)	Non-*H*30 (n = 7)	*H*30 non-Rx (n = 10)	*H*30Rx (n = 31)	Non-*H*30 *vs*. *H*30^d^	*H*30-non-Rx *vs*. Rx
**Adhesins**	*papAH*	16 (33)	3 (43)	0 (0)	13 (42)	0.67	0.02
	*papC*	17 (35)	5 (71)	0 (0)	12 (39)	0.08	0.02
	*papEF*	17 (35)	5 (71)	0 (0)	12 (39)	0.08	0.02
	*papG*^*e*^	15 (31)	3 (43)	0 (0)	12 (39)	0.66	0.02
	*papGII*	13 (27)	1 (14)	0 (0)	12 (39)	0.66	0.02
	*papGIII*	1 (2)	1 (14)	0 (0)	0 (0)	0.15	NA
	*sfa/foc*	1 (2)	1 (14)	0 (0)	0 (0)	0.15	NA
	*iha*	41 (85)	3 (43)	8 (80)	30 (97)	**< 0.01**	0.14
	*afa/dra*	13 (27)	1 (14)	0 (0)	12 (39)	0.66	0.02
	*hra*	17 (35)	4 (57)	0 (0)	13 (42)	0.23	0.02
**Toxins**	*hlyD*	11 (23)	4 (57)	0 (0)	7 (23)	0.04	0.16
	*hlyF*	7 (15)	3 (43)	1 (1)	3 (10)	0.05	1.0
	*cnf1*	7 (15)	0 (0)	0 (0)	7 (23)	0.57	0.16
	*cdtB*	1 (2)	1 (14)	0 (0)	0 (0)	0.15	NA
	*sat*	41 (85)	2 (29)	9 (90)	30 (97)	**< 0.01**	0.43
**Siderophores**	*iroN*	6 (13)	3 (43)	0 (0)	3 (10)	0.03	0.56
	*fyuA*	46 (96)	5 (71)	10 (100)	31 (100)	0.02	NA
	*iutA*	45 (94)	5 (71)	9 (90)	31 (100)	0.05	0.24
**Protectins and invasins**	*kpsMII* ^*f*^	40 (83)	5 (71)	5 (50)	30 (97)	0.33	**< 0.01**
	*K1*	1 (2)	1 (14)	0 (0)	0 (0)	0.15	NA
	*K2*	25 (52)	2 (29)	0 (0)	23 (74)	0.24	**< 0.01**
	*K5*	8 (17)	0 (0)	3 (30)	5 (16)	0.58	0.38
	*K15*	1 (2)	1 (14)	0 (0)	0 (0)	0.15	NA
	*iss*	8 (17)	4 (57)	1 (10)	3 (9.7)	0.01	1.0
	*traT*	38 (79)	4 (57)	8 (80)	26 (84)	0.15	1.0
	*cvaC*	4 (8)	1 (14)	1 (10)	2 (6)	0.48	1.0
	*ibeA*	6 (12.5)	6 (83)	0 (0)	0 (0)	**< 0.01**	NA
**Other**	*ompT*	46 (96)	5 (71)	10 (100)	31 (100)	0.02	NA
	*malX*	44 (92)	3 (43)	10 (100)	31 (100)	**< 0.01**	NA

^a^Genes detected in all isolates: *fimH*, *yfcV*, and *usp*.

^b^Genes not detected in any isolate: *papGI/I’*, *sfaS*, *focG*, *afaE8*, *bmaE*, *gafD*, *F17*, *and clpG* (adhesins); *pic*, *vat*, *tsh*, *astA* (toxins); *ireA* (siderophore); *kpsMTIII* and *rfc* (protectins); and *H7 fliC*, *clbN*, and *clbB* (other).

^c^P values obtained by chi^2^ test or Fisher’s test as appropriate. Statistically significant values are in bold.

^d^*H*30 included the non-Rx and Rx subsets.

^e^Isolates with at least one *papG* allelic variant or that are PCR-positive with generic *papG* primers.

^f^Isolates with at least one *kpsII* variant. NA: Not applicable.

Aggregate VG profiles were defined based on presence/absence of the studied VGs. The *kpsMII* and *pap* operons were considered to be present if any of the corresponding genes or their allelic variants were detected. Isolates were assigned to virotypes (A, B, C, D1-5, and E) as described by Mora *et al*. based on presence/absence of specific VGs: *ibeA* (invasion of brain endothelium), *iroN*, *sat* (secreted autotransporter toxin), *afa/dra*, *papGII*, *papGIII*, *cnf1* (cytotoxic necrotizing factor 1), *hlyD* (ɑ-haemolysin), *cdtB* (cytolethal distending toxin), and K1 (group II capsule variant) [15]. Isolates not corresponding to a defined virotype were classified as “other”.

### Murine sepsis model

Isolates were tested for *in vivo* virulence using a modified protocol of an established murine subcutaneous sepsis model [26]. Experimentation guidelines of the Veterans Affairs Medical Centre of Minneapolis were followed in the conduct of the mouse model experiments under animal use protocol 120603, as approved by the local Institutional Animal Care and Use Committee (IACUC). Mice were kept in ventilated cages (maximum 10 mice per cage) with food and water *ad libitum* and following natural cycles of light and darkness. Briefly, female pathogen-free outbred Swiss-Webster mice (mean age 60 days, mean weight 23g) were inoculated subcutaneously in the nape of the neck with approximately 10^9^ CFU/mL log-phase bacteria in 0.2 mL saline. Mouse health was assessed twice daily for 3 days post-challenge. Mice were classified daily as to maximal illness severity, which ranged from 1 (healthy) to 5 (dead), with scores 2 (barely ill), 3 (moderately ill) and 4 (severely ill) in between. Scores were based on external exam of the mice and their behaviour (i.e., redness around the eyes or nose, cleaning habits, huddling, and movement). The exams were performed concurrently by two experienced investigators who were unaware of the characteristics of the challenge strains, and then reached a consensus regarding the score. Each particular characteristic was not scored, rather, all were considered jointly to reach the final illness score. All mice that reached score 4 were euthanized to minimize suffering. All surviving mice were euthanized at the end of the study according to the local IACUC guidelines, using carbon dioxide inhalation. All efforts were taken to minimize suffering of the mice at all times, and no analgesic or anaesthetic was needed. No unexpected deaths were observed. Each experimental series included a positive control strain (urosepsis isolate CFT073; 80%-100% of mice dead by 3 days post-challenge) and a negative control strain (laboratory strain MG1655; no perceptible mouse illness or lethality) [26]. Each test strain was assessed initially in 5 mice, followed by another 5 mice if the initial testing did not yield a consistent result (i.e., lethality or survival for 4 or 5 of the initially challenged mice).

Daily illness severity scores for the mice challenged with a given isolate were averaged to give the isolate's mean illness severity score (ISS). The variables used for comparisons of virulence potential included overall mean ISS (as a continuous variable), rapid lethality (i.e., mean illness severity score ≥ 4, indicating that all mice were severely ill or dead by day 1 post-inoculation).

### Statistical analysis

Continuous variables were described as medians and interquartile ranges (IQR), and were compared using the Mann-Whitney or Kruskal-Wallis test, as appropriate. Dichotomous variables were described using frequencies and percentages, and were compared using a chi-square test or Fisher’s exact test, as appropriate. The criterion for statistical significance was p < 0.05. The Bonferroni correction method was applied when multiple comparisons were performed. Data were analysed with software STATA version 11 (Stata Statistical Software: Release 11. College Station, TX: StataCorp LP). A heatmap representing presence/absence of the studied VGs in relation to strain characteristics (subclone, ExPEC status, ISS, virotype and virulence profile) was performed using RStudio software version 1.0.44 (RStudio Team 2016, RStudio: Integrated Development for R. RStudio, Inc., Boston, MA) with the pheatmap package.

## Results

### Study isolates

**Fig 1:**
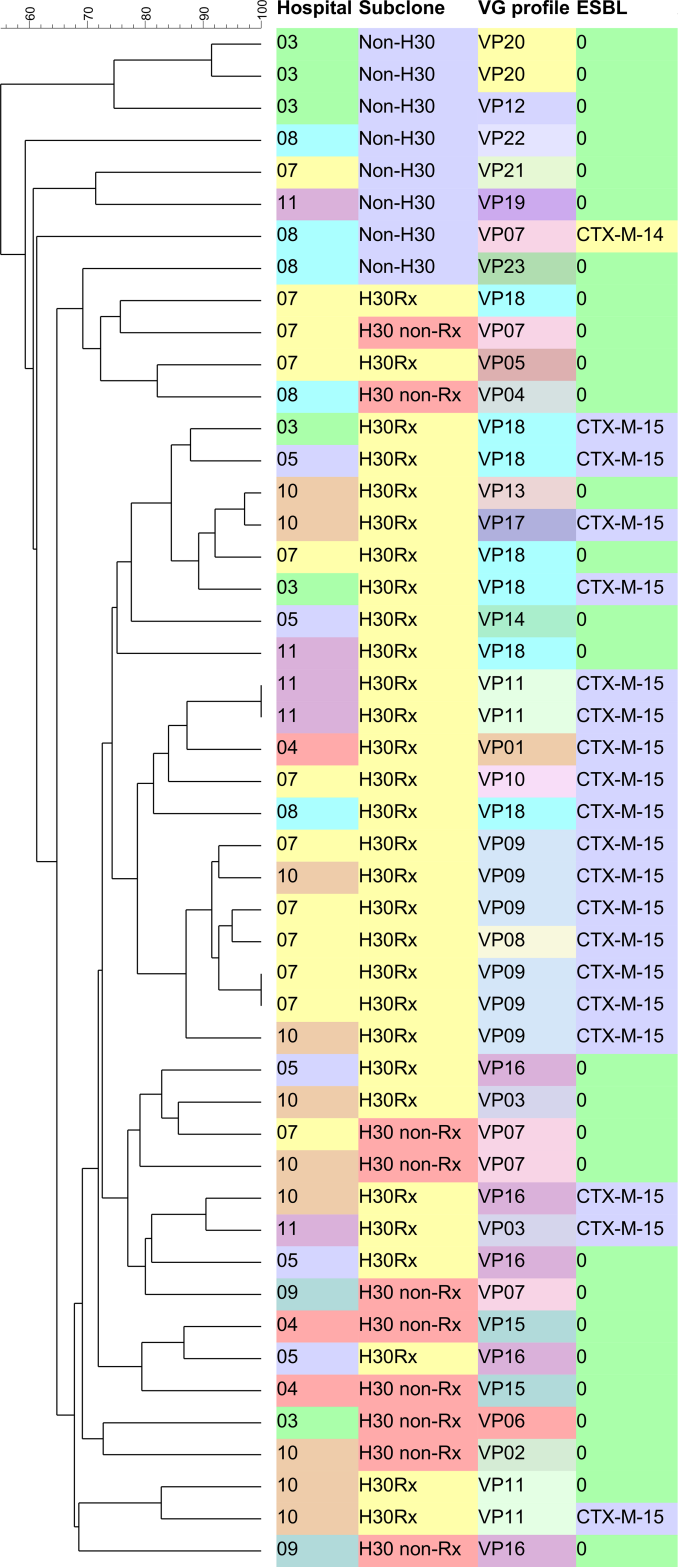
Dendrogram of the 48 ST131-*E*. *coli*. Hospitals: 03-Hospital Gregorio Marañón (Madrid); 04-Hospital del Mar (Barcelona) 05-Hospital Marqués de Valdecilla (Santander); 07-Hospital Ramón y Cajal (Madrid); 08-Hospital de la Santa Creu i San Pau (Barcelona); 09-Hospital Virgen Macarena (Sevilla); 10-Complejo Sanitario de Pontevedra (Pontevedra); 11-Hospital Mutua Tarrassa (Barcelona). VG profile: Virulence genes profile. VP: Virulence profile.

Of the 48 ST131 bloodstream isolates, 31 (65%) were *H*30Rx, 10 (21%) were *H*30 non-Rx and 7 (15%) isolates belonged to the non-*H*30 subclone. Twenty-one isolates (44%) were ESBL producers, of which all but one belonged to the *H*30Rx subclone (Fig 1). By O-type PCR, 47 isolates (98%) were O25b and one (2%) was O16. The dendrogram showed that ST131 subclones were evenly distributed across participant hospitals without a clonal overrepresentation (Fig 1).

### Virulence genes, scores, and ExPEC status

Of the 49 VGs tested, 32 (65%) were detected in at least 1 isolate, at frequencies ranging from 2% to 100% (Table 1) and all isolates encoded at least 8 VGs. All isolates contained *fimH*, *usp*, and *yfcV* (putative fimbrial subunit). Apart from these, the most prevalent VGs of each functional category were *iha* (adhesin, 85%), *sat* (toxin, 85%), *fyuA* (siderophore, 96%), *kpsMII* (protectin, 83%), and *ompT* (other, 96%).

The three ST131 clonal subsets (*H*30Rx, *H*30 non-Rx, and non-*H*30) differed significantly for the prevalence of certain VGs (Table 1). Whereas *ibeA* (83%) and *iss* (57%) were found mainly in non-*H*30 isolates (*versus* 0% and 10% in *H*30-positive isolates respectively, p ≤ 0.01), *malX* (100%), *sat* (95%) and *iha* (93%), were detected more commonly among the *H*30 isolates (*versus* 43%, 29% and 43% respectively among the non-*H*30 isolates, p < 0.01). Moreover, the VGs *kpsII* and K2 differed significantly in prevalence between the *H*30Rx and *H*30 non-Rx isolates (97% *versus* 50% and 74% *versus* 0% respectively, p < 0.01). Also of note, the *H*30 non-Rx isolates did not encode any VG of the *pap* operon and in fact the only adhesins found in this subclone were *fimH*, *yfcV* and *iha*.

Overall, VG scores ranged from 8 to 17 (median 12, IQR 10.5–14). Scores differed by subclone, with medians (IQR) of 12 (11–15) for *H*30Rx, 11 (10–14) for non-*H*30 isolates, and 10 (10–10) for *H*30 non-Rx (p < 0.01 for the three-group comparison). ESBL production was associated with higher VG score with median (IQR) VG score of 13 (12–15) in ESBL producers *versus* 10 (10–13) in non-ESBL producers (p <0.01).

Forty (83%) study isolates fulfilled molecular criteria for ExPEC, including all 31 *H*30Rx isolates (100%) and 71% (5/7) of non-*H*30 isolates but only 40% (4/10) of *H*30 non-Rx isolates (*versus H*30Rx: p < 0.01) (Table 2). ExPEC isolates showed higher VG scores than non-ExPEC isolates (median 12 *versus* 10: p < 0.01).

**Table 2:** Virulence genes scores, ExPEC status, and experimental virulence according to subclone and virotype.

	Virulence-related genotypes			Mouse sepsis model outcomes	
ST131 Subclone	Virotypes (no. isolates)	Median VG^a^ score (IQR^d^)	ExPEC (row %)	Median ISS^b^ (IQR^d^)	Rapid lethality (row %)
Non-*H*30	C (n = 1)	10 (0)	0 (0)	4.4 (0)	1 (100)
	D3 (n = 1)	11 (0)	1 (100)	4.5 (0)	1 (100)
	D4 (n = 1)	10 (0)	0 (0)	1.7 (0)	0(0)
	Other^c^ (n = 4)	13.5 (3.5)	4 (100)	4.5 (1.4)	3 (75)
	Total (n = 7)	11 (3)	5 (71)	4.4 (2.4)	5 (71)
*H*30 non-Rx	C (n = 9)	10 (0)	4 (44)	2.9 (1.6)	3 (33)
	Other (n = 1)	8 (0)	0 (0)	1.5 (0)	0 (0)
	Total (n = 10)	10 (0)	4 (40)	2.75 (1.9)	3 (30)
*H*30Rx	A (n = 12)	12 (1)	12 (100)	4.5 (0.75)	9 (75)
	B (n = 2)	15 (0)	2 (100)	3.4 (1.8)	1 (50)
	C (n = 5)	11 (0)	5 (100)	4.1 (2)	3 (60)
	E (n = 7)	15 (0)	7 (100)	3.9 (1.7)	3 (43)
	Other (n = 5)	13 (0)	5 (100)	4.7 (0.3)	5 (100)
	Total (n = 31)	12 (4)	31 (100)	4.3 (1.5)	21 (68)

^a^VG: virulence gene.

^b^ISS: illness severity score.

^c^”Other”: isolates that did not correspond with a defined virotype.

^d^ IQR: Interquartile range

### Virotypes and virulence profiles

Overall, 79% (38/48) of isolates corresponded with a previously described virotype, most commonly virotype C (31%) or A (25%) (Table 3). Virotype C occurred within all three subclones (16% of *H*30Rx, 90% of *H*30 non-Rx, and 14% of non-*H*30 isolates), while the other virotypes were found exclusively in certain subclones (Table 2). Moreover, virotypes differed significantly for VG scores (p < 0.01 overall) with virotype B and E showing the highest VG score with median of 15 (IQR 15–15) (Table 3).

**Table 3:** Prevalence of characteristics (column %) and median scores according to virotypes.

	Virotype						
Charac.^b^	A (n = 12)	B (n = 2)	C (n = 15)	D3 (n = 1)	D4 (n = 1)	E (n = 7)	Other^a^ (n = 10)
ExPEC	12 (100)	2 (100)	9 (60)	1 (100)	0 (0)	7 (100)	9 (90)
VG score (IQR)^c^	12 (1)	15 (0)	10 (1)	10.5 (0)	10 (0)	15 (0)	13 (1)
ISS (IQR)^d^	4.5 (0.75)	3.4 (1.8)	3.8 (2)	4.5 (0)	1.7 (0)	3.9 (1.7)	4.6 (0.4)
RL^e^ (col. %)	9 (75)	1 (50)	7 (47)	1 (100)	0 (0)	3 (43)	8 (80)

^a^Other: isolates did not correspond with a defined virotype.

^b^Characteristics: For comparisons across all virotypes, the only statistically significant difference involved virulence gene scores (p < 0.01).

^c^ VG score: Median virulence gene score (interquartile range).

^d^ ISS: Median illness severity score (interquartile range).

^e^ Rapid lethality.

**Fig 2:**
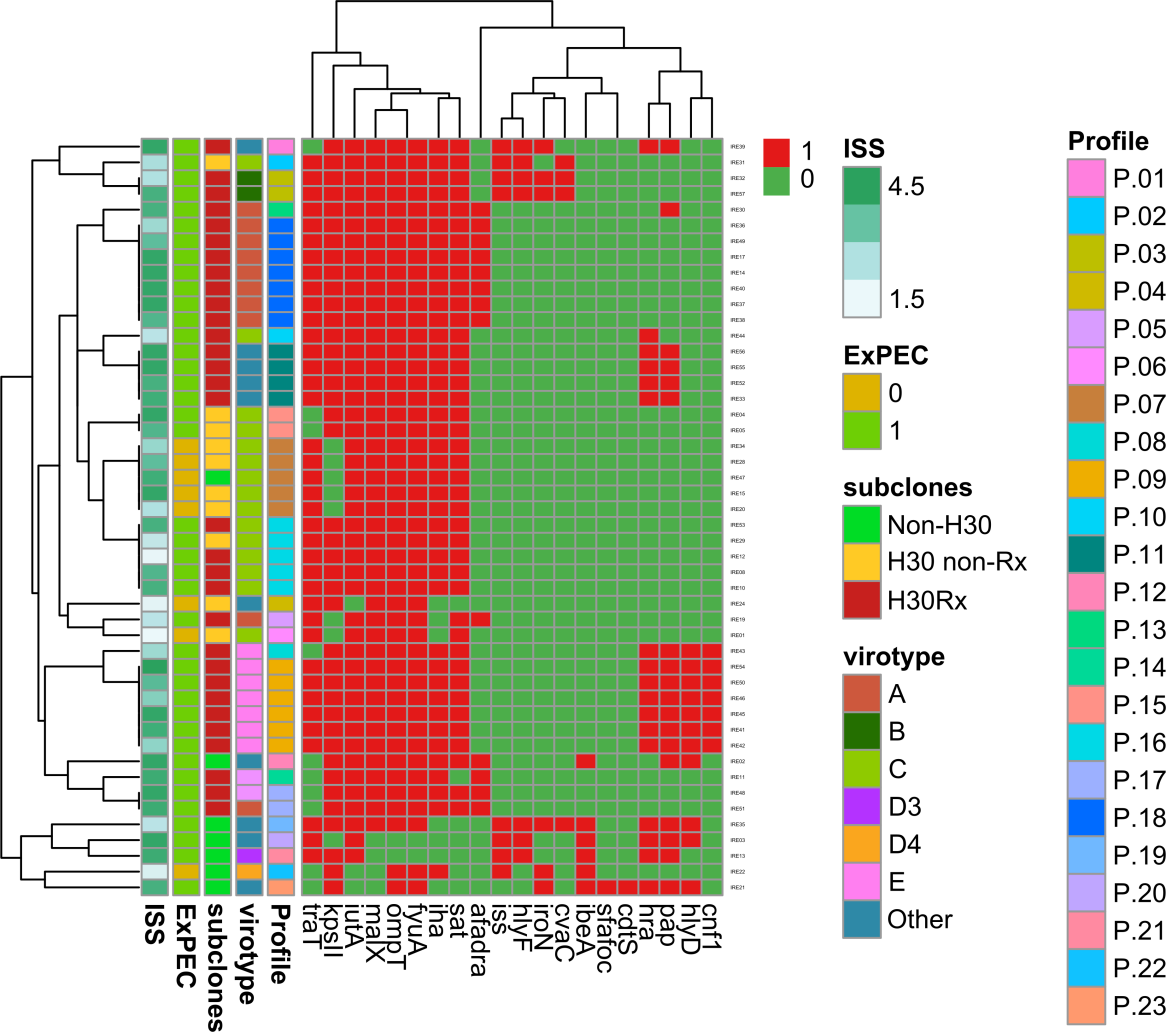
Heatmap with the virulence genes tested of the 48 *Escherichia coli* ST131 study isolates. Only variably present virulence genes are shown, with all *pap* and *kps* variants counted as one; *pap*: isolates positive for any of the *pap* operon genes tested, i.e., *papAH*, *papC*, *papEF*, *papG* (with alleles I, I’, II, III), *kps II*: isolates positive for any of the *kps II* variants tested, i.e., *kpsMII*, *K1*, *K2*, *K5* and *K15*. Virulence genes present or absent in all isolates are not shown. Red indicates presence and green indicates absence of gene. “Other” category (virotype column): a virotype could not be assigned. ISS: illness severity score. RL: rapid lethality. Profile (P): Aggregated virulence genes profile.

Twenty-three different VG profiles (arbitrarily numbered P1 to P23) were observed (Fig 2). Diversity of profiles varied by clonal subset, that is, whereas collectively the 31 *H*30Rx isolates showed only 12 total profiles, and the 10 *H*30 non-Rx isolates only 6 total profiles, each of the 7 non-*H*30 isolates had a unique profile.

### Murine sepsis model

Overall, the 48 study isolates showed a high level of morbidity and lethality in the murine sepsis model: the median ISS was 4.3 (IQR 2.8–4.7), and 29 (60%) were rapidly lethal. ESBL producers showed greater illness severity (median ISS, 4.4 for ESBL producers, *versus* 4.1 for non-ESBL producers: p = 0.04) and were more often rapidly lethal (71% *versus* 52%, p = 0.17).

By clonal subset, the *H*30Rx and non-*H*30 isolates exhibited the greatest illness severity (median ISS, 4.3 and 4.4, respectively), and *H*30 non-Rx isolates the lowest (median ISS, 2.75; *versus* other isolates: p = 0.04). Likewise, most *H*30Rx and non-*H*30 isolates exhibited rapid lethality (71% and 68%, respectively), as compared with only 30% of *H*30 non-Rx isolates (*H*30Rx *versus H*30 non-Rx: p = 0.06) (Table 2).

The illness severity score was also greater in ExPEC isolates (median ISS, 4.3 [ExPEC] *versus* 2.7 [non-ExPEC]: p = 0.03). In contrast, VG scores and aggregate VG profiles were not significantly associated with any sepsis model outcome, and among individual VG, only K2 was associated with higher ISS (median 4.45 in K2-positive isolates *versus* median 3.35 in K2-negative isolates, p < 0.01). Moreover, virotype A and D3 isolates showed the highest median ISS (4.5) although neither of them had the highest VG score and there was no significant overall association between virotype and these virulence outcomes (Table 2).

## Discussion

In this study, we assessed 48 Spanish *E*. *coli* ST131 bloodstream isolates for VG content (expressed as individual VGs, ExPEC status, VG scores, VG profiles, and virotypes) and ST131 subclone status, and compared these bacterial characteristics with virulence in a murine sepsis model (expressed as ISS, and rapid lethality).

We found no significant association of *in vivo* virulence with VG scores, extended VG profiles, or virotypes. Moreover, among the 49 VG studied, the only one that was associated with a higher ISS was K2, which encodes certain capsular antigen variants K2. Although ESBL production seemed possibly to be associated with higher VG score and ISS, almost all (20/21) ESBL-producing isolates belonged to the *H*30Rx subclone, so this association could result from confounding factors. Also, the K2 gene was detected more often among *H*30Rx isolates (74%) than in the other isolates (17%), which also points to subclone classification as the reason for this association. In contrast, the ST131 subclones and ExPEC *versus* non-ExPEC isolates differed significantly for *in vivo* virulence. These differences suggest that the bacterial traits determining experimental virulence in this murine model are distributed differentially between ST131 clonal subsets, and between ExPEC *versus* non-ExPEC ST131 isolates, but do not necessarily include the VGs studied here or segregate reliably by virotype. Moreover, these data could indicate that the recent epidemic expansion of the *H*30 ST131 subclone–at least its large non-Rx component–must have explanations other than hyper-virulence, at least as measured in this murine sepsis model. Indeed, in this study the *H*30 non-Rx isolates showed a noticeable lack of VGs (mainly adhesins), specially compared with the phylogenetically related *H*30Rx subclone.

Overall, the present ST131 isolates exhibited high *in vivo* virulence, as described previously for other ST131 isolates [5,15]. However, we found *in vivo* virulence to vary considerably both between and within the different ST131 subclones, consistent with the findings of Johnson *et al* [9]. This variability in experimental virulence between and within ST131 subsets may explain why no consistent lethality differences have been found between ST131 isolates generally and non-ST131 isolates [9,15].

The *H*30 subset within ST131, with its Rx and non-Rx components, is the leading cause of ST131 expansion [20]. Interestingly, in the present comparison of experimental virulence and VG content, the *H*30Rx isolates more closely resembled the non-*H*30 isolates than they did the *H*30 non-Rx isolates, which conflicts with the underlying phylogenetic relationships. Subclone differentiation was not considered in previous reports from several authors that described variability among experimental virulence in ST131 strains and the lack of correlation between virulence gene content and experimental virulence [15,16,21]. Our results indicate that subclones should be studied individually to assess correlation between VG and virulence.

Olesen *et al*. suggested that the expansion of ST131, and of its *H*30Rx subclone in particular, could be due more to a specific subset of genes (not necessarily encoding exclusively antibiotic resistance or virulence) than to total number of VGs [20]. Recent whole genome analysis has pointed to the acquisition of certain accessory genes as the starting point for the clade C (or *H*30 subset) expansion [4]. Our results further refine these hypotheses, since although the *H*30Rx isolates exhibited some of the highest VG and lethality scores, the *H*30 non-Rx (i.e., clade C1) isolates had lower VG scores and were significantly less virulent than even the non-*H*30 isolates. Our results suggest that these subclone-specific differences in experimental virulence may result from genetic differences between subclones that involve sequences other than classic VGs. The acquisition of virulence traits, therefore, may not be the evolutionary event that drove the clade C expansion. The distinctive accessory traits of non-Rx *H*30 isolates, although seemingly unlikely to confer enhanced virulence, may enhance the organism's ability to colonize the host or to perform other non-directly pathogenic functions that favour clonal expansion, and so could be called more appropriately fitness traits. The limited number of strains in our study, however, does not allow for robust conclusions, and further studies clearly are needed.

A previous study that compared the virulence of ST131 and non-ST131 clinical isolates by using the same murine sepsis model as used here showed that the presence of certain genes (i.e., *papAH*, *kpsMII*, *papGIII*, *vat*, K1, and *clbB/N*), rather than ST131 status, was associated with higher lethality [9]. Conversely, a recent study that compared urinary and bloodstream isolates virulence in an insect larva model found an association of ST131 status with higher experimental virulence [7]. Notably, however, that model has been shown to yield results that do not correspond with those obtained in the murine sepsis model [27]. Here, in comparing different ST131 isolates in the murine sepsis model, despite considerable overall variability in virulence genotypes and virulence outcomes we found no significant associations between specific virulence genes and experimental virulence with the exception of the K2 gene.

Our inability to identify associations of virotype with virulence outcomes in the murine sepsis model also differs from previous findings [10,28]. Here, although virotype C was most common, it did not exhibit exceptionally high illness severity, lethality, or VG scores, whereas in two previous studies by different groups of investigators it was associated with increased mouse lethality [15,28]. We found that ST131 subclone and ExPEC status were better predictors of experimental virulence than was virotype, with the association of ExPEC status and higher lethality possibly being due to all *H*30Rx isolates qualifying as ExPEC. The inconsistent *in vivo* virulence differences observed here among virotypes suggest that the VG combinations that define the established virotypes do not reliably classify ST131 isolates according to their experimental virulence in the murine sepsis model.

This study's main limitations included the relatively small number of isolates (especially representing the *H*30 non-Rx subclone), which restricted statistical power. Moreover, almost half of the isolates were provided by two of the eight participating hospitals, although minimal evidence of intra-hospital clonality was found. However, few studies have addressed experimental virulence in relation to ST131 subclone classification, as our study does. Moreover, our main objective was to describe the findings and generate new hypothesis for subsequent more in-depth testing. Another possible limitation was the focus on bloodstream isolates from Spain, which restricted generalizability. This murine model does not address completely the pathogenesis of bloodstream infections such as previous colonization or persistence. Moreover, although this murine sepsis model is well-established, the use of presence/absence of virulence genes as a marker, without considering levels of expression, could affect the in vivo virulence results.

Our study’s main strengths were the representative study population (which was drawn broadly from different geographic areas of Spain), the inclusion of both ESBL-producing and non-producing isolates, the wide range of tested VGs, the attention to key ST131 subclones, and the use of a well-established murine sepsis model.

### Conclusion

We found that experimental virulence in the murine sepsis model varies considerably among ST131 isolates, even within previously defined virotypes, whereas subclone and, to a lesser extent, ExPEC status identifies isolates with similar levels of experimental virulence. The comparatively high lethality of *H*30Rx isolates suggests that as-yet undefined sequences associated with this subclone might be responsible for its ability to cause severe infections. In contrast, the comparatively low virulence of non-Rx *H*30 isolates point away from virulence as an explanation for this subgroup's impressive epidemic emergence. Further study is needed to understand the basis for ST131's successful expansion and to find markers of experimental lethality that also can predict clinical outcome in infected patients.
